# Temporal Interference Electrical Stimulation for Neuropsychiatric Disorders: Mechanisms, Applications, and Translational Perspectives

**DOI:** 10.3390/ijms27094023

**Published:** 2026-04-30

**Authors:** Yaqi Zhang, Yue Tong, Xiangyang Zang, Yaqiong Zhao, Feng Wang, Xueliang Shang, Yanxue Xue

**Affiliations:** 1 Henan Key Laboratory of Medical Tissue Regeneration, Henan Medical University, Xinxiang 453003, China; 15560119889@163.com (Y.Z.); 17737512875@163.com (Y.Z.); wfeng100@126.com (F.W.); 2 Hebei Key Laboratory of Mental Health and Brain Science, School of Psychology and Mental Health, North China University of Science and Technology, Tangshan 063210, China; 15140187493@163.com (Y.T.); shangxueliang@ncst.edu.cn (X.S.); 3 Department of Neurobiology, School of Basic Medical Sciences, National Institute on Drug Dependence, Peking University, Beijing 100191, China; zxy1191908531@163.com

**Keywords:** temporal interference electrical stimulation, non-invasive deep neuromodulation, neuropsychiatric disorders, neuromodulation, synaptic plasticity

## Abstract

Neuropsychiatric disorders are characterized by complex etiologies, widespread involvement of brain regions, and pronounced clinical heterogeneity, with core pathological mechanisms closely associated with abnormal activity in deep brain structures and their functional networks. Although current pharmacological therapies and conventional neuromodulation techniques have shown therapeutic benefits in certain conditions, they are generally limited by insufficient stimulation depth or the risks associated with invasive procedures. Temporal interference (TI) electrical stimulation has recently emerged as a non-invasive deep neuromodulation technique that generates low-frequency difference-envelope fields through high-frequency carrier signals, thereby enabling relatively precise modulation of deep brain regions while maintaining favorable safety and tolerability. This technique provides a novel technical pathway for precision intervention in neuropsychiatric disorders. In this review, we summarize the principles and technical characteristics of TI stimulation and highlight its recent applications in mood and stress-related disorders, cognitive impairment and neurodegenerative diseases, movement disorders, addiction, and disorders associated with dysregulated neural excitability. We integrate its potential mechanisms across multiple levels, including neural oscillations, deep–cortical network synchronization, reward and motivational circuits, synaptic plasticity and structural remodeling, excitatory-inhibitory balance, and gene and epigenetic regulation. Current evidence suggests that TI stimulation can modulate electrophysiological activity and may engage molecular and network-level processes relevant to functional improvement, although durable clinical benefits remain to be established. Although clinical translation remains challenged by parameter optimization, interindividual variability, and long-term safety evaluation, advances in computational modeling, multimodal neuroimaging, and closed-loop stimulation strategies are expected to facilitate its development. Overall, TI stimulation represents a promising non-invasive deep neuromodulation approach for mechanistic investigation and precision treatment of neuropsychiatric disorders.

## 1. Introduction

Neuropsychiatric disorders represent a major group of diseases worldwide that cause functional impairment, reduced quality of life, and substantial socioeconomic burden, encompassing a broad range of clinical conditions including mood disorders, anxiety disorders, Alzheimer’s disease, Parkinson’s disease, substance addiction, and epilepsy [1,2]. Epidemiological evidence indicates that these disorders are highly prevalent, prone to relapse, and often persistent, imposing sustained challenges on individual health, family structures, and public health systems [2,3]. Clinically, neuropsychiatric disorders are characterized by chronic progression, co-occurrence of multiple symptoms, and marked heterogeneity, involving diverse functional domains such as cognition, emotional regulation, executive control, reward processing, and motor behavior, and their pathological basis extends beyond dysfunction of single neurons or neurotransmitter systems [4,5].

Extensive basic and clinical studies indicate that the core pathological mechanisms of neuropsychiatric disorders involve abnormal functional connectivity among deep brain regions and disrupted patterns of neural network information processing [6,7]. For example, major depressive disorder is commonly associated with impaired reward and executive control circuits between the nucleus accumbens and the prefrontal cortex, leading to anhedonia and motivational deficits [8]; Parkinson’s disease is characterized by pathological enhancement of beta-band oscillations within the basal ganglia-thalamocortical circuits, resulting in bradykinesia and rigidity [9]; and addiction-related disorders are closely linked to abnormal reinforcement learning, altered synaptic plasticity, and amplified reward prediction errors in the mesolimbic dopamine system [10,11]. Collectively, these findings support the view that neuropsychiatric disorders represent systemic disorders of deep brain networks and their dynamic regulation [12].

Although current therapeutic strategies continue to evolve, including pharmacological treatments, psychological interventions, and various neuromodulation techniques, they remain insufficient for restoring normal physiological activity in the deep pathological neural circuits involved in neuropsychiatric disorders [13]. Pharmacological therapies generally target broad neurotransmitter systems and lack circuit-level precision, and their long-term use is frequently associated with tolerance, reduced adherence, and systemic side effects [14]; psychological therapies are beneficial in some patients but show limited efficacy in moderate-to-severe or treatment-resistant conditions [15]. Consequently, achieving modulation of disease-related deep neural circuits with high spatial specificity and functional selectivity remains an unresolved challenge in the treatment of neuropsychiatric disorders.

With the increasing recognition of the concept of “brain network disorders,” traditional therapeutic paradigms centered on individual neurons and neurotransmitters are gradually being replaced by a new framework focused on modulation of functional networks and neural circuits [16]. Temporal interference (TI) electrical stimulation has recently emerged as a non-invasive deep neuromodulation approach with considerable potential for precision intervention in neuropsychiatric disorders [17]. This technique applies multiple sets of high-frequency sinusoidal currents with slightly different frequencies on the scalp, generating low-frequency difference-envelope electric fields within deep brain structures and thereby selectively modulating deep neuronal activity while markedly reducing stimulation of superficial cortical regions [18]. Owing to its distinctive physical mechanism, TI enables highly focal modulation of specific deep brain regions and functional circuits without electrode implantation and is therefore regarded as a promising alternative or complement to non-invasive DBS [19]. With advances in computational modeling, electrode configuration optimization, and multimodal neuroimaging, TI has been increasingly applied to key deep brain regions involved in emotion, memory, motor control, and reward processing, showing encouraging effects in multiple neuropsychiatric disease models [20].

Based on the above background, this review aims to systematically present the physical principles, technical characteristics, and safety profile of temporal interference electrical stimulation, summarize current applications and available evidence across different neuropsychiatric disorders, and provide an integrated analysis of its multi-level biological mechanisms, including modulation of neural oscillations, remodeling of neurotransmitter systems, and regulation of synaptic plasticity. In addition, we discuss the technical challenges and practical limitations encountered during clinical translation and outline future perspectives of TI in precision neuromodulation and individualized treatment of neuropsychiatric disorders, providing a theoretical basis and reference framework for further research and clinical application.

## 2. Principles of Temporal Interference Electrical Stimulation and Technical Considerations

**Figure 1:**
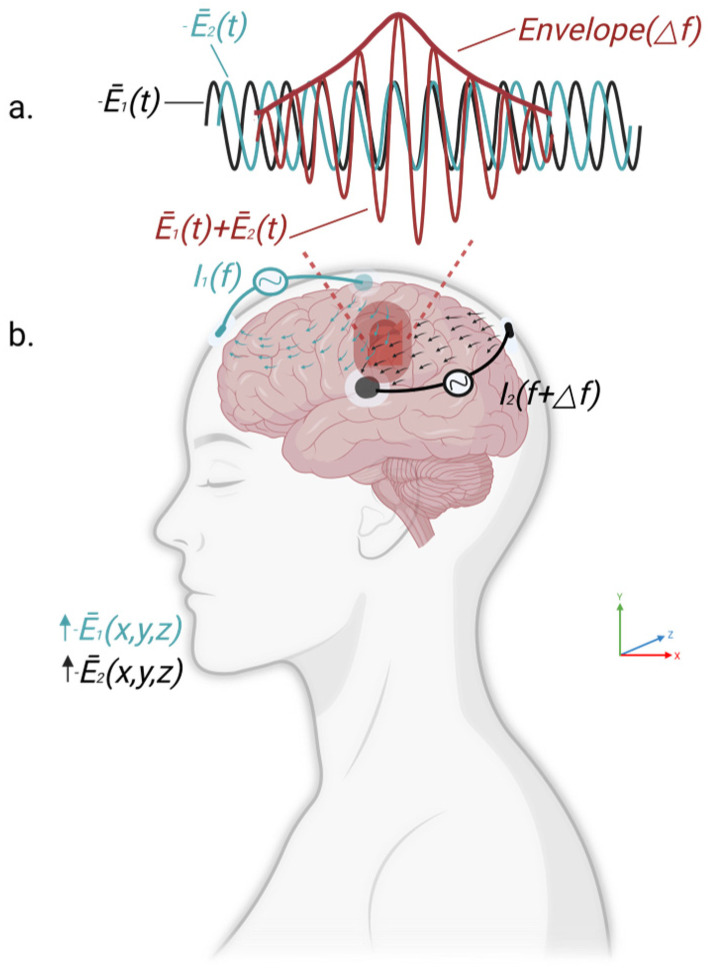
Physical principles and spatial characteristics of temporal interference (TI) electrical stimulation. (**a**) Schematic representation of the generation of a localized interference field. Two high-frequency alternating currents, I1(f) and I2(f + Δf), are delivered into the brain. While the individual electric fields (E1 and E2) oscillate at frequencies too high to effectively recruit neurons, their vector superposition creates an amplitude-modulated total electric field (E1 + E2) with a low-frequency envelope at the offset frequency (Δf). (**b**) Spatial distribution of TI stimulation in the human brain. The configuration of two pairs of electrodes allows the modulation envelope to reach deep-seated brain structures while minimizing the activation of the overlying superficial cortex. The coordinate system (x, y, z) indicates the spatial orientation of the induced electric field vectors.

TI stimulation is a novel non-invasive neuromodulation technique proposed in recent years, aiming to achieve non-invasive stimulation of deep brain regions [18]. This method was first introduced by Grossman et al. in 2017 and involves the application of two sets of high-frequency alternating currents with closely spaced frequencies through different scalp electrodes. The high-frequency currents can readily penetrate the cortex and superficial tissues but have minimal direct activating effects on neurons. As illustrated in Figure 1, when these two currents converge within deep brain regions, they interfere with each other, generating a low-frequency modulated electric field with a frequency equal to the difference between the two carrier frequencies [21]. This electric field can effectively activate deep neurons, thereby overcoming the limitations of conventional transcranial electrical stimulation in terms of stimulation depth and field dispersion. This technique enables neuromodulation to shift from superficial to deeper brain structures while reducing discomfort and side effects associated with direct cortical stimulation [22].

From a neurophysiological standpoint, neuronal membranes display low-pass characteristics in response to high-frequency (>1 kHz) electric field variations. Due to membrane capacitance, neurons respond minimally to the rapidly alternating components of high-frequency sinusoidal currents, whereas they are relatively more sensitive to amplitude-modulated signals at frequencies far below the kilohertz carriers [23]. Accordingly, under TI stimulation, although high-frequency currents can penetrate the cortex without directly activating superficial neurons, in deep brain regions where the two currents intersect, the resulting amplitude-modulated envelope may bias membrane polarization and promote neuronal responses. This feature provides a plausible basis for the noninvasive electrophysiological modulation of specific deep brain structures [17].

By adjusting the relative positions of the two electrode sets, the frequency difference (Δf), and the phase difference (Δφ), spatial focusing and directional control of the interference region can be achieved [24]. The location of the interference region can be precisely regulated by modifying electrode geometry, whereas tuning of the envelope frequency allows parameterized customization to different neural circuit characteristics, such as δ, θ, β, and γ rhythms [25]. A key advantage of this technique is its capacity to combine deep penetration with high spatial selectivity, thereby addressing the technical limitations of conventional transcranial stimulation techniques [26]. This may improve the potential for noninvasive modulation of deep reward-related structures, including the nucleus accumbens and the ventral tegmental area [27].

Recent finite element model (FEM)-based simulation studies have provided further evidence for the focusing capability and controllability of TI stimulation. In realistic three-dimensional human head models, multipair TI stimulation employing more than two electrode pairs was shown to improve the focalized delivery of stimulation to deep brain regions compared with the conventional two-pair configuration [28]. In addition, individually customized TI stimulation further demonstrated that optimization of electrode configurations and injection currents in different head models can enhance focal modulation of a deep target while reducing unwanted stimulation in neocortical regions [29].

Moreover, recent computational modeling studies have introduced multi-frequency interference and spatiotemporal modulation strategies to further improve deep focusing capability and targeting flexibility [26]. For instance, incorporating dynamic phase control algorithms into multi-electrode systems enables focal point mobility (“electric field scanning”) at the millisecond time scale, allowing sequential stimulation of multiple regions without physical movement of the electrodes [30]. These advances provide an important theoretical basis for the development of future TI-based closed-loop brain stimulation systems [31,32].

In typical temporal interference electrical stimulation, two or more high-frequency currents (e.g., 2000 Hz and 2010 Hz) are applied to the scalp to create a low-frequency envelope at the difference frequency in deeper brain areas. The spatial distribution, focality, and depth of this modulation field depend on several factors: carrier frequency, current intensity, electrode layout, current ratio, and tissue conductivity. Recent computational and measurement studies show that optimizing electrode placement and balancing current can increase envelope modulation in deep targets while partly limiting cortical stimulation [33]. For example, intracranial recordings in non-human primates showed that symmetrically arranged bilateral electrode pairs produced stronger TI power in central deep regions, whereas altering the current ratio between the two sides shifted the distribution of TI power, indicating that both electrode montage and current balancing can shape deep-field focusing and steerability [34]. In the same study, TI power increased approximately linearly with stimulation intensity and remained relatively stable across the tested carrier-frequency range, suggesting that, under these experimental conditions, stimulation intensity and montage design may be more influential than carrier frequency in determining effective field strength [34]. Recent human evidence further indicates that TI can be applied in patients with implanted deep brain stimulation electrodes, with local field potentials recorded via externalized DBS leads, while individualized finite-element models explicitly incorporated the implanted electrodes. In addition, phantom measurements in that study did not reveal focal charge enhancement near the implanted lead, although the potential influence of conductive implants on current flow and local electric-field distribution still warrants careful evaluation [35]. Nevertheless, the achievable spatial resolution and effective stimulation volume remain constrained by skull-related current shunting, tissue heterogeneity, individual anatomical variability, and the accuracy of electric-field modeling [33].

These physical and technical constraints also have important implications for the biological interpretation of TI effects, particularly regarding whether modulation occurs predominantly through suprathreshold activation or through subthreshold network-level mechanisms [36,37,38].

## 3. Applications of Temporal Interference Electrical Stimulation in Neuropsychiatric Disorders

With continued progress in electric-field modeling and basic research, TI stimulation has begun to be explored in animal models and early-stage human studies. Available evidence suggests that non-invasive TI targeting deep functional hubs-such as the hippocampus, striatum/basal ganglia-related targets, and subthalamic nucleus-can induce measurable effects on memory-related processing, reinforcement learning, motor-network activity, and pathological electrophysiological biomarkers [30,39]. This section reviews disease-specific applications of TI, highlighting experimental and clinical functional outcomes and their corresponding targets, to provide a factual foundation for subsequent mechanistic analysis.

### 3.1. Mood and Stress-Related Disorders

Mood- and stress-related disorders—including major depressive disorder (MDD), anxiety disorders, and post-traumatic stress disorder (PTSD)—are among the most prevalent and clinically burdensome categories of neuropsychiatric diseases [40,41]. A large body of neuroimaging and neurophysiological evidence suggests that their core pathology arises not from a single regional abnormality but from disrupted functional coordination within key prefrontal-limbic circuits [42]. In particular, reduced reactivity of the NAc to positive reward stimuli, together with impaired medial prefrontal cortex (mPFC) function in emotional regulation and executive control, contributes to anhedonia, diminished motivation, and blunted affect [43]. At the same time, excessive amygdala activation during threat-related cue processing enhances negative emotional experiences, anxiety responses, and stress sensitivity [44,45]. Such multi-node, multi-pathway dysfunction undermines adaptive emotional regulation and represents a key neural basis for the persistence of depressive and anxiety symptoms.

In this context, TI stimulation, owing to its non-invasive deep neuromodulatory properties, provides a novel approach for targeting key structures involved in emotional regulation. Existing evidence suggests that TI may enhance neural responsiveness to positive emotional and reward stimuli by modulating the NAc and its associated reward pathways, thereby mitigating anhedonia and reduced motivation commonly observed in depression [46]. In parallel, deep brain stimulation (DBS) of the amygdala—particularly its basal nucleus—has been demonstrated to alleviate anxiety and improve PTSD symptoms by modulating fear extinction-related circuits, highlighting the amygdala as a critical regulatory node in mood- and stress-related disorders [47,48]. Given TI’s ability to modulate deep brain regions non-invasively, it may theoretically influence emotion-related nuclei, including the amygdala, thereby offering a non-invasive intervention option for anxiety disorders and PTSD [49].

TI, by virtue of its ability to reach deep targets with relatively high spatial selectivity, may support top-down emotional regulation through modulation of prefrontal and deep subcortical circuits [26]. Neuroimaging evidence suggests that TI can alter connectivity within task-relevant networks, including increased frontoparietal coupling during working memory and altered striatal–frontal interactions during reinforcement learning, indicating its potential relevance for cognitive control and emotion-related regulation [50,51]. Given the central role of the PFC in emotion regulation, threat inhibition, and extinction, such network-level effects may also be relevant to anxiety- and trauma-related symptoms, including fear extinction and memory-updating processes implicated in PTSD [52].

Overall, a potential advantage of TI in mood and stress-related disorders lies in its capacity to non-invasively engage deep circuit-relevant targets that are difficult to access with conventional surface-focused stimulation [30,39]. Early human evidence remains limited but includes preliminary symptomatic improvement with NAc-targeted tTIS in bipolar depression, alongside ongoing clinical efforts to test sgACC-targeted tTIS in major depressive disorder; by contrast, amygdala-related applications remain largely theoretical at present [46,49,53]. In contrast to conventional non-invasive stimulation approaches that act mainly on superficial cortical regions, TI shows stronger functional relevance for deep emotion-regulating centers and may provide a novel intervention avenue, especially for treatment-resistant patients who respond poorly to pharmacological and traditional neuromodulatory therapies.

### 3.2. Cognitive Impairment and Neurodegenerative Diseases

In Alzheimer’s disease (AD) and its prodromal stage, mild cognitive impairment (MCI), cognitive and memory deficits are not attributable to localized degeneration of a single brain region but instead reflect dysfunction within hippocampal-centered memory circuits and their coordinated interactions with cortical networks. Task-based EEG studies have demonstrated that normal working memory processing is typically accompanied by robust post-stimulus modulation in the theta/alpha bands, whereas in AD/MCI populations, reduced theta-band modulation is more stable and predictive of working memory performance, suggesting that aberrant theta oscillatory regulation represents a key electrophysiological hallmark of cognitive decline [54]. Functional imaging studies further reveal that during hippocampus-dependent tasks, patients with high-burden MCI and AD show significantly attenuated hippocampal activity, together with disrupted deactivation patterns in medial parietal regions of the default mode network (DMN) (e.g., the precuneus and posterior cingulate cortex), which are inversely coupled with hippocampal task components, indicating that memory impairment reflects a dynamic imbalance at the hippocampal-cortical network level [55].

From a neurodynamic systems perspective, cognitive processing relies on temporal coordination and rhythmic synchronization across brain regions within specific frequency bands. Extensive evidence indicates that beta/gamma-band synchronization contributes to multiple cognitive processes, including attention, information integration, and working memory, whereas in several brain disorders, including AD, reduced local and long-range synchrony commonly coexists with multidimensional cognitive deficits, suggesting that aberrant rhythmic synchronization plays a key role in disease mechanisms [56]. Accordingly, targeted modulation of rhythm disturbances centered on the hippocampus and its associated networks has become an important research direction for cognitive intervention.

Based on the above deep-circuit–neural oscillation pathological features, TI has been proposed as a non-invasive neuromodulation approach with the potential to act on deep memory-related circuits. Existing proof-of-concept studies indicate that under specific parameter settings and electrode configurations, TI can exert relatively selective modulatory effects on the hippocampus, accompanied by altered hippocampal activity and improved memory performance during task-based fMRI, supporting the feasibility of modulating deep memory networks [32,57].

In neurodegenerative disease models, preliminary evidence has linked TI effects to interactions among neural oscillations, network-level activity, and behavior. In an APP/PS1 mouse preprint study, TI stimulation was associated with changes in neurotransmitter-related and excitatory/inhibitory balance-related markers at the targeted site, including increased GABA levels and reduced NMDA/GluN1-related signaling, together with alterations in gamma rhythms, cross-frequency coupling, brain connectivity, and behavioral performance [58]. These findings provide preliminary support for the possibility that TI may influence cognitive function through coordinated modulation of molecular, oscillatory, and network-level processes. Nevertheless, TI remains at an early translational stage, and systematic studies are still needed to optimize stimulation parameters, implement individualized modeling, and evaluate long-term effects [30,39,57].

### 3.3. Movement Disorders and Basal Ganglia Diseases

Parkinson’s disease (PD) is a neurodegenerative disorder primarily characterized by bradykinesia, rigidity, and resting tremor, with its core pathological mechanisms closely associated with abnormally enhanced beta-band neural oscillations within the basal ganglia-thalamocortical circuit [59,60]. Extensive evidence indicates that excessive synchronization of beta oscillations suppresses normal motor initiation and execution processes and constitutes an important neurophysiological basis of PD motor symptoms [61]. Although conventional DBS can effectively suppress pathological beta oscillations by directly targeting the subthalamic nucleus or the internal segment of the globus pallidus, thereby significantly improving motor symptoms, it relies on invasive surgical procedures and is associated with risks such as infection, hemorrhage, and hardware-related complications, which limits its broader application across patient populations [62]. Accordingly, exploring an alternative neuromodulation strategy that combines deep-targeting capability with non-invasive characteristics is of important clinical significance.

TI stimulation, as an emerging non-invasive deep neuromodulation technique, offers a new possibility for intervening in PD-related basal ganglia dysfunction. By applying multiple high-frequency currents on the scalp and generating a low-frequency envelope in deep brain regions, TI can be configured to target deep structures such as the subthalamic nucleus (STN), thereby modulating basal ganglia circuit activity. Existing evidence indicates that STN-targeted TI/TIS can reduce pathological beta-band oscillatory activity in patients with PD, supporting its potential relevance for motor symptom modulation [35,63]. These findings suggest that TI exhibits the potential to simulate the electrophysiological modulation pattern of deep brain stimulation (DBS) without the need for electrode implantation.

At the preclinical level, TI stimulation has demonstrated positive effects in PD animal models. Relevant studies have shown that TI can improve motor initiation, gait stability, and limb coordination, with therapeutic effects in specific motor behavior dimensions comparable to those of conventional DBS. At the same time, the deep focusing property of TI markedly reduces direct excitability effects on the cortex and other non-target brain regions, thereby decreasing potential adverse effects and enhancing stimulation safety and tolerability [30,39,63].

Beyond animal experiments, recent preliminary clinical studies have further supported the potential of TI stimulation in patients with PD. One study reported that 130 Hz TI targeting the right globus pallidus internus significantly reduced bradykinesia and tremor, with more pronounced improvements observed in the contralateral limbs, which is consistent with the principle of crossed motor pathway innervation [64]. These findings indicate that TI, as a non-invasive deep brain stimulation approach, has good feasibility and safety in clinical applications, and may provide a new therapeutic option for patients who are not suitable for surgery or are cautious about undergoing conventional DBS [35,39].

In summary, TI stimulation demonstrates broad potential in the treatment of PD and related basal ganglia disorders. Its effects in reducing pathological beta oscillations and improving motor symptoms suggest that TI may, to some extent, compensate for the limitations of conventional DBS in terms of invasiveness and risk [35,39,64,65]. With further optimization of stimulation parameters, target selection, and individualized modeling strategies, TI may be combined with pharmacological therapy and other rehabilitation interventions to provide PD patients with safer, more precise, and more individualized comprehensive treatment options [39,65].

### 3.4. Addiction and Impulse Control Disorders

Addiction and impulse control disorders are tightly associated with pathological reinforcement of the mesolimbic–striatal reward system and persistent impairment of prefrontal cognitive control functions [66,67,68]. At the circuit level, the dopaminergic pathway from the VTA to the NAc is considered the core circuit underlying reward learning and motivational drive, whereas the dorsal striatum, particularly the dorsolateral striatum (DLS), plays a key role in repetitive behaviors and habit formation [69,70]. In the addicted state, abnormal activity within these circuits, especially aberrant bursting of dopaminergic neurons and instability of phasic signaling, is thought to drive pathological reward-seeking behavior, impulsive decision-making, and relapse vulnerability [71,72,73].

By generating a low-frequency difference envelope in deep brain regions, TI stimulation provides a new technical pathway for non-invasive modulation of key addiction-related circuits [18,20]. Given its potential targeting capability for deep structures, TI is thought to modulate neural excitability and network coupling in the NAc, VTA, and related striatal regions without markedly interfering with superficial cortical activity, thereby potentially influencing abnormal neural activity associated with addictive behaviors [74]. This feature makes TI a powerful tool for studying and intervening in the neural mechanisms of addiction [20].

Behavioral studies in animal models provide direct evidence for the application of TI stimulation in addiction intervention [74]. Multiple experiments have shown that following TI stimulation, animals exhibit a significant reduction in impulsive behavior in conditioned place preference (CPP) and drug-seeking behavior tests, together with a marked decrease in sensitivity to drug-related cues and preference behavior [74,75,76]. These behavioral changes are highly consistent with TI-induced modulation of activity in reward-related brain regions, suggesting that TI can effectively intervene in the neural basis of addictive behaviors.

From a target-specific modulation perspective, TI may influence dopaminergic signaling and striatal–frontal interactions, thereby affecting reward prediction and reinforcement learning [51,77,78]. In addition, TI may strengthen prefrontal-striatal regulation by modulating neuroplasticity at relevant input sites [79,80]. This coordinated modulation of reward and control systems may help reduce relapse risk and support long-term behavioral change.

Compared with conventional pharmacological and behavioral interventions, TI offers potential advantages including non-invasiveness, favorable safety, and good physiological compliance [20,32,39]. These features support its potential as an adjunctive circuit-based approach for refractory addiction and impulse control disorders [74,81].

### 3.5. Disorders Associated with Imbalanced Neural Excitability

Epilepsy is characterized by excessive network-level excitability and pathological synchronization, with seizures commonly originating from focal brain regions and propagating along distributed neural networks. Disruption of the excitatory/inhibitory (E/I) balance is widely regarded as a key pathogenic mechanism, as abnormal inhibitory interneuron migration or impaired synaptic development during early life can render neural circuits persistently susceptible to hyperexcitability [82,83,84]. The hippocampus and limbic system, due to their high epileptogenic propensity, frequently constitute core components of epileptogenic foci and pathological networks [85,86]. Notably, these deep structures are also critically involved in emotional and memory processing; for example, the basolateral amygdala–ventral hippocampus pathway plays an essential role in anxiety regulation and spatial memory [87,88,89]. Therefore, modulation of deep epileptogenic networks requires a delicate balance between suppressing abnormal discharges and avoiding disruption of key cognitive-emotional circuits [85,86].

Although anti-seizure medications remain the first-line treatment, approximately one-third of patients progress to drug-resistant epilepsy (DRE) [90,91]. In this population, resective surgery is often limited by multifocal seizure foci or involvement of eloquent functional regions, whereas invasive neuromodulation strategies such as DBS, RNS, and VNS can serve as alternative options but are accompanied by implantation-related complications and potential risks of cognitive side effects [92,93]. Therefore, developing a non-invasive technique that reduces invasiveness while still enabling targeted modulation of deep epileptogenic networks represents a clear and unmet clinical need [92,93].

TI generates low-frequency envelope modulation in the brain through the superposition of kilohertz carrier currents with a small frequency difference, thereby enabling subthreshold modulation of targeted regions [94,95]. In epilepsy applications, the direction-tunable property of the TI electric field is particularly critical: animal studies have shown that when the field orientation is aligned with the hippocampal axonal axis, relatively focal stimulation of structures such as hippocampal CA3 can be achieved without the need for penetrating electrodes, and this alignment significantly influences the threshold of evoked events [94]. This non-penetrating stimulation mode may also complement sEEG by covering brain regions that are difficult to access because of anatomical or safety constraints, thereby potentially reducing the number of implanted electrodes and the associated risks [94,96].

Recent studies in sEEG-implanted patients have provided preliminary direct human evidence for the therapeutic potential of TI [97]. In patients with drug-resistant mesial temporal lobe epilepsy, hippocampus-targeted TI (Δf ≈ 130 Hz) was associated with significant reductions in interictal epileptiform discharges and pathological high-frequency oscillations, attenuation of the interregional propagation of abnormal activity, and a short-term suppressive effect persisting after stimulation offset [97]. These findings further suggested that TI primarily exerts its effects through subthreshold amplitude-modulated electric fields, thereby partially mitigating the limitation of conventional transcranial stimulation in which superficial effects tend to dominate [97]. Combined with individualized imaging-based modeling and electric field simulation, TI provides a promising technical pathway for non-invasive and relatively precise modulation of deep epileptogenic networks; however, its actual benefits for seizure frequency, long-term cognitive-emotional outcomes, and therapeutic stability still require confirmation in larger samples and long-term follow-up studies [24,32].

In summary, TI has demonstrated cross-system neuromodulatory advantages across multiple neuropsychiatric disorders [20,39]. Improvements in emotional, cognitive, motor, reward-related, and neural excitability domains appear to stem from direct modulation of abnormal activity within deep functional circuits. Although the current evidence base remains limited, it has established a preliminary foundation for further clinical translation of TI [30]. With ongoing advances in signal parameter optimization, electrode technology, and individualized modeling strategies, the therapeutic role of TI in neuropsychiatric disorders is expected to be further strengthened and validated [24,30,98].

### 3.6. Challenges and Potential of TI in Human Clinical Applications

Although TI stimulation is theoretically regarded as having unique advantages, its real-world translational application remains constrained by multiple factors, particularly those related to stimulation parameter selection and the influence of inter-individual variability on electric field distribution. Existing evidence indicates that TI can effectively modulate neural activity in deep brain regions in animal models, supporting its feasibility in terms of spatial targeting [25,34]. However, considerable uncertainty persists regarding its application in the human brain. For example, inter-individual differences in electric field intensity and spatial distribution may compromise stimulation precision at intended deep targets [24,99]. In addition, current research is still dominated by animal experiments and computational modeling, while the number of human studies remains limited, which is insufficient to allow a comprehensive evaluation of its clinical feasibility [39,100].

In the context of the above limitations, TI, as a novel non-invasive neuromodulation approach, still shows a development potential worthy of attention. Existing evidence suggests that TI can, to some extent, improve motor performance and enhance functional coupling between cortical and basal ganglia networks [51,78]. For example, previous work has shown that TI can facilitate motor skill acquisition and alter striatal activity or striatal-frontal interactions, while more recent studies have also reported facilitatory effects in specific motor tasks such as vertical jump performance [51,78,101]. In addition, TI has shown a positive trend in working memory modulation, although its effect magnitude remains relatively limited compared with some existing techniques [50,101,102].

**Figure 2:**
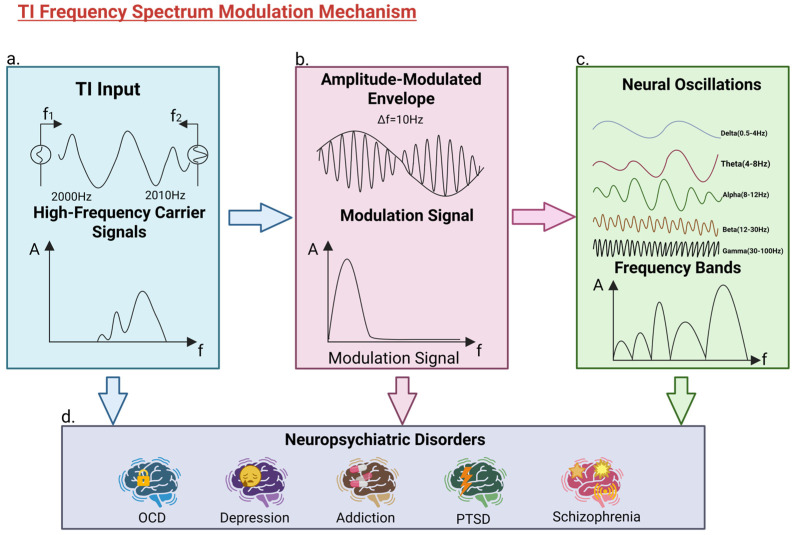
(**a**) TI signal input consisting of two high-frequency carrier currents (f1 = 2000 Hz and f2 = 2010 Hz). (**b**) Extraction of the amplitude-modulated envelope at the offset frequency (Δf = 10 Hz) through temporal interference, shifting the effective modulation from kilohertz carrier components to a biologically relevant stimulation frequency. (**c**) Interaction between the modulation signal and endogenous neural oscillations across multiple frequency bands, including Delta, Theta, Alpha, Beta, and Gamma waves. (**d**) Clinical application of TI stimulation for various neuropsychiatric disorders, such as obsessive–compulsive disorder (OCD), depression, addiction, post-traumatic stress disorder (PTSD), and schizophrenia, by re-tuning aberrant oscillatory patterns.

Therefore, although the clinical use of TI is currently constrained by limited evidence and individual variability, its potential role in deep brain neuromodulation should not be overlooked. Future studies need to assess its efficacy and safety in larger human cohorts and to further explore individualized parameter optimization strategies, in order to support its stepwise translation toward clinical application [24,100]. The cascade from high-frequency signal input to the modulation of endogenous neural oscillations and its subsequent clinical implications is summarized in Figure 2.

## 4. A Unified Mechanistic Framework of TI: Systemic Effects from Molecules to Networks

**Figure 3:**
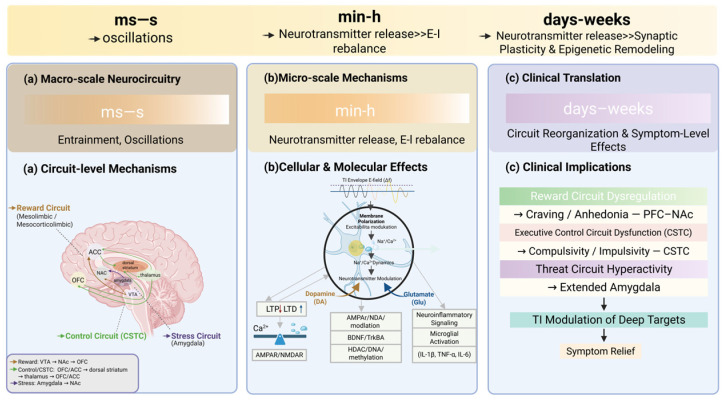
Multiscale mechanisms of temporal interference (TI) stimulation. TI targets deep-brain circuits, including mesolimbic, CSTC, and threat-related pathways, to induce oscillatory entrainment, neurotransmitter rebalancing, and synaptic plasticity-related changes (e.g., LTP/LTD and BDNF-associated mechanisms), thereby facilitating functional circuit restoration and symptom improvement across neuropsychiatric disorders such as addiction, OCD, and anxiety disorders. Different colors are used to distinguish conceptual modules and pathways across macro-scale neurocircuitry, micro-scale cellular mechanisms, and clinical translation; they are intended for visual differentiation rather than quantitative comparison.

Although TI has shown functional improvements across multiple neuropsychiatric disease models and early clinical studies, these effects are unlikely to be explained by modulation of a single brain region or an isolated molecular target. Instead, accumulating evidence suggests a multi-level, cross-time-scale mode of action, in which modulation of deep brain activity may be associated with coordinated changes spanning neural oscillations, functional circuits, synaptic plasticity, and downstream molecular processes [30]. Based on the disease-oriented evidence summarized above, this section integrates the potential mechanisms of TI, including modulation of neural oscillations and deep functional circuits [32,37], synaptic structural and functional remodeling [80], and potential plasticity-related neurochemical adaptations [77]. By linking relatively rapid electrophysiological effects with subsequent plasticity-associated changes, this unified framework may help explain the broad applicability of TI and its potential long-term therapeutic value. The multiscale mechanistic cascade underlying TI stimulation is summarized in Figure 3.

### 4.1. Neural Oscillation Modulation and Reshaping of Deep-Cortical Network Synchrony

Neural oscillations are a core dynamic feature of brain information processing and interregional communication. Temporal coordination across frequency bands supports sensory integration, cognitive processing, and behavioral output [103]. Substantial evidence indicates that neuropsychiatric disorders do not result from dysfunction in a single brain region, but are closely associated with network-level disruptions of frequency-specific oscillatory patterns [104]. Different disorders exhibit relatively specific abnormalities across the oscillatory spectrum. For example, excessive β-band oscillations within the basal ganglia-cortical circuit in PD are tightly linked to motor inhibition [59], whereas impaired θ–γ cross-frequency coupling in AD and MCI is associated with deficits in memory encoding and information integration [105]. In depressive disorders, large-scale network dysfunction is implicated in impaired emotion regulation and cognitive control, whereas in addiction-related conditions, dysregulated prefrontal–striatal interactions are linked to craving, impaired executive control, and relapse vulnerability [106,107].

TI provides a new technical pathway for targeted modulation of neural oscillations in deep brain regions through its difference-frequency envelope mechanism [37]. Unlike conventional transcranial stimulation, which primarily affects superficial cortical oscillations, TI is thought to generate low-frequency effective electric fields in deep structures, thereby enabling relatively selective modulation of specific neural rhythms. This feature positions TI as a neuromodulation strategy centered on oscillatory control and oriented toward brain network remodeling [20]. By acting on deep rhythm-generating sources, TI may influence temporal coordination across distributed brain regions, with potential implications for large-scale network integration [37,103].

In PD-related models, TI targeting the subthalamic nucleus has been reported to suppress pathologically elevated β-band synchrony, thereby reducing abnormal inhibition of motor execution pathways and promoting improvements in motor function [35]. This finding provides mechanistic support for the non-invasive modulation of basal ganglia pathological oscillations by TI. In parallel, memory-related studies and hippocampal-targeting work suggest that TI can focally modulate hippocampal activity and may support episodic memory performance, although direct evidence specifically linking TI to restoration of hippocampal θ-γ cross-frequency coupling in AD remains limited [20,108]. Together, these findings across different disease models support a potential mechanism whereby TI may reshape deep neural oscillatory patterns and help restore the rhythmic basis of large-scale brain network function.

Beyond motor and cognitive disorders, increasing attention has been directed toward the modulatory effects of TI on emotion- and motivation-related networks. In MDD and addictive disorders, dysfunction within prefrontal-nucleus accumbens and related corticostriatal circuits is considered a key neural mechanism underlying emotional dysregulation, impaired impulse control, and abnormal reward processing [107,109]. Emerging evidence suggests that TI can enable targeted modulation of deep-cortical networks and may influence cross-regional oscillatory coordination; however, direct evidence specifically demonstrating enhanced long-range synchrony between the pre-frontal cortex and nucleus accumbens remains limited [20,110]. This potential modulation of deep–cortical network coupling provides an oscillatory framework for understanding the possible applicability of TI across multiple neuropsychiatric disorders.

It is worth emphasizing that the effects of TI are unlikely to reflect a simple enhancement or suppression of neural activity within an isolated brain region. Rather, TI may promote a shift of pathological brain networks toward a more adaptive functional state by modulating cross-regional oscillatory coupling, phase synchrony, and information flow [30,37]. This network-centered mode of regulation supports the conceptualization of TI as a form of network-oriented neuromodulation [30]. By non-invasively acting on deep rhythm-generating sources, TI may partially overcome some limitations of conventional neuromodulation techniques in stimulation depth and spatial specificity, thereby providing a theoretically coherent mechanistic basis for interventions in PD, MDD, addiction, and other neuropsychiatric disorders [18,32].

### 4.2. Reward and Motivation Circuit Modulation: Dopamine System and Behavioral Output Rebalancing

The core pathological mechanisms of mood and addiction disorders are commonly linked to functional dysregulation of the midbrain dopamine system during reward learning, motivational drive, and behavioral selection [111]. Within this circuitry, the VTA-NAc pathway is regarded as a key neural substrate for regulating hedonic experience, reward prediction, and goal-directed behavior [112]. In pathological states, this system often exhibits aberrant temporal dynamics of dopamine signaling, an increased bias toward reinforcement learning, and attenuated responsiveness to natural rewards, thereby giving rise to behavioral phenotypes such as anhedonia, heightened craving, and impulsive decision-making [109,112].

TI stimulation enables noninvasive modulation of reward and motivation circuits by generating a difference-frequency envelope in deep brain regions [30]. Studies indicate that TI can directly influence neural activity in structures such as the NAc and the VTA, with minimal effects on superficial cortical activity, and can show bidirectional modulatory effects under certain conditions [18,20,113]. In reward-related experimental work, TI has been shown to modulate striatal phasic dopamine signaling, providing preliminary mechanistic support for the possibility that TI may influence anhedonia- and motivation-related processes in mood and addiction disorders [46,77].

In addiction models, the effects of TI stimulation on the reward system appear to follow a distinct functional direction. Evidence from a morphine-conditioned place preference model suggests that TI stimulation can reduce preference for drug-paired contexts and attenuate conditioned reward-related behavioral bias [74]. These findings suggest that, across different pathological conditions, TI may influence reward-related behavioral output by modulating reinforcement-related processes and motivational salience attribution [111].

At the molecular and synaptic levels, the downstream mechanisms of TI remain incompletely characterized; current evidence more directly supports modulation of dopaminergic signaling and network-level plasticity, whereas the precise involvement of D1/D2 receptor balance, the cAMP-PKA-CREB cascade, and glutamatergic synaptic remodeling remains to be systematically validated [30]. In addition, emerging human work suggests that TI can modulate neural systems relevant to cognitive control, although direct evidence specifically demonstrating enhanced ventral striatum–prefrontal functional connectivity and reduced impulsive decision-making remains limited [39,114].

Notably, current evidence suggests that the effects of TI stimulation are less consistent with a simple unidirectional excitation or inhibition model and may instead reflect more selective and state-dependent modulation of neural function across distributed brain networks [38,115]. This characteristic supports the relatively high theoretical safety and adaptability of TI for interventions in mood and addiction disorders [100,116].

Overall, these findings provide a mechanistic framework supporting the application of TI stimulation in mood and addiction disorders, particularly underscoring its potential as a complementary approach to pharmacological and behavioral treatments. Future studies will be necessary to clarify the condition-dependent modulation of the dopaminergic system by TI and to characterize its differential effects across clinical subtypes, thereby promoting its translation toward more precise and individualized clinical applications [116].

### 4.3. Synaptic Plasticity and Structural Remodeling

Synaptic plasticity and neural structural remodeling represent fundamental biological mechanisms underlying both the persistence of neuropsychiatric symptoms and the consolidation of therapeutic effects over time [117]. As a neuromodulation technique with the potential to target deep brain regions, temporal interference (TI) stimulation may influence synaptic processes by modulating neuronal excitability and the temporal structure of neural activity [30]. Current evidence suggests that TI can enhance neuroplasticity-related processes, including synaptic transmission efficiency, dendritic spine density, neurotransmitter release, and the expression of synapse-related proteins, thereby providing a biological basis for sustained functional improvement [118].

At the synaptic level, TI-related enhancement of neuroplasticity has been associated with increased expression of synapse-related proteins, enhanced excitatory synaptic transmission, and other plasticity-related changes, whereas the more specific receptor-level glutamatergic mechanisms are still inferred mainly from the broader synaptic plasticity literature rather than directly demonstrated in TI-specific studies [118,119]. Such changes in synaptic transmission dynamics are thought to contribute to memory encoding and emotional regulation. Notably, hippocampus-targeted TI in humans has been shown to focally modulate hippocampal activity and improve episodic memory accuracy, supporting a link between TI-induced modulation of deep neural systems and behaviorally relevant plasticity-related outcomes [120].

At the level of synaptic structure, animal studies further support a role for TI stimulation in promoting neural structural remodeling. Experimental findings indicate that TI can induce both structural and functional plasticity, including increased dendritic spine density and other neuroplasticity-related changes, providing experimental support for its potential influence on learning- and memory-related brain regions [80,118]. Dendritic spines, as the principal structural substrates of excitatory synapses, are widely regarded as important markers of synaptic plasticity, and their density and morphology closely reflect plasticity states [121,122]. These observations suggest that TI may produce effects extending beyond short-term neural modulation and may contribute to more sustained improvement of impaired neural networks through synaptic structural remodeling [80,118].

In addition, as an electric field-based neuromodulation technique, TI stimulation may play a role in synaptic maintenance and structural stability through the regulation of neurotrophic factors and their associated signaling pathways. Previous studies on electrical stimulation more broadly suggest that neurotrophic signaling pathways, including BDNF/TrkB- and ERK/MAPK-related mechanisms, may participate in activity-dependent synaptic maintenance, synaptic stability, and neuronal survival [118,123]. By analogy, these findings provide indirect support for the possibility that TI may engage neurotrophic factor-related pathways, although direct TI-specific evidence remains limited [124].

Compared with traditional transcranial alternating current stimulation (tACS), TI stimulation may offer an advantage in achieving greater spatial selectivity within deep brain regions [18,37]. This property may allow TI to more selectively engage deep pathological structures, such as the hippocampus, while limiting broader cortical co-stimulation, thereby supporting its translational potential [18,32]. Although key stimulation parameters-including carrier frequency combinations, stimulation intensity, and duration-remain to be systematically optimized, existing studies have provided initial evidence of sustained effects across multiple neuropsychiatric disease models, establishing an important mechanistic basis for future clinical applications [30,118].

### 4.4. Inhibitory and Excitatory Neurotransmitter Regulation: Restoration of E/I Dynamic Balance

The preservation of normal brain function depends on a finely regulated balance between excitatory neurotransmitters, primarily glutamate, and inhibitory neurotransmitters, mainly γ-aminobutyric acid (GABA), a state commonly referred to as E/I ratio homeostasis [125]. Substantial evidence indicates that disruption of this balance is a common pathological feature across diverse neuropsychiatric disorders, including epilepsy, schizophrenia, depressive disorders, anxiety disorders, and addictive behaviors [126,127]. Within this framework, neuromodulatory interventions capable of restoring E/I dynamic balance are regarded as having broad potential for disease intervention [126].

TI stimulation represents a non-invasive neuromodulation approach with the potential to influence network activity through spatially selective and cell-type-dependent effects [18,38]. Current evidence suggests that TI may modulate excitatory–inhibitory interactions at the network level and therefore holds potential therapeutic relevance for disorders characterized by dysregulated excitability [20,38].

In epilepsy-related studies, TI stimulation has been shown to reduce pathological hippocampal activity, including interictal epileptiform discharges, pathological high-frequency oscillations, and their propagation patterns [95,97]. Beyond this acute suppression, these findings suggest that TI may help stabilize hippocampal network excitability [97].

Beyond epilepsy, TI stimulation may also hold theoretical potential for modulating E/I-related network dynamics in mood-related disorders [20,126]. Through its capacity for relatively selective engagement of deep brain circuits, TI may influence activity within neural systems relevant to emotional regulation [18,32]. Emerging evidence further suggests that TI can modulate activity in key cortico-striatal circuits, with studies reporting effects on striatal activity, phasic dopamine signaling, and reinforcement-related processing [51,77,78]. Rather than acting on a single neurochemical pathway, these effects are more likely to arise from membrane polarization, nonlinear ion-channel responsiveness, and network-level interactions that shape neural oscillations and circuit dynamics [30,37,38]. In addition to modulating neurotransmitter levels, TI stimulation may also potentially influence activity-dependent synaptic plasticity [32]. The low-frequency envelope resulting from the interference of high-frequency electric fields may modulate neuronal firing synchrony and shape network oscillations—mechanisms that are thought to play a pivotal role in activity-dependent synaptic plasticity, such as long-term potentiation (LTP) and long-term depression (LTD) [30,128]. These synaptic plasticity mechanisms represent fundamental cellular substrates for learning, memory, and behavioral adaptation. They may also contribute to the therapeutic effects of TI stimulation in neuropsychiatric disorders, although direct evidence specific to TI remains limited [129]. Additionally, receptor-dependent pathways—particularly NMDA receptor-mediated glutamatergic transmission and GABAergic inhibitory signaling—may also be involved in modulating neural circuit dynamics during TI stimulation, although this possibility has not yet been directly established for TI [124].

In addition to neuronal mechanisms, non-neuronal cells, such as astrocytes and microglia, may also contribute to the outcomes of electrical stimulation. These support and immune cells affect processes like synaptic transmission and neuroinflammation, and their activity is sensitive to external electric fields. The key implication is that both neuronal and non-neuronal elements must be considered to fully assess the broad effects of neuromodulation [130]. Further clarifying the involvement of non-neuronal cells, previous studies have shown that external electric fields can induce intracellular Ca^2+^ signaling in astrocytes, leading to the propagation of astrocytic Ca^2+^ waves and the release of gliotransmitters such as ATP and glutamate [131]. These signals can activate purinergic receptors (P2X and P2Y) expressed on astrocytes and microglia, thereby modulating synaptic transmission and neuronal excitability. Through neuron-glia interactions, i.e., bidirectional communication between neurons and glial cells, TI stimulation may indirectly influence synaptic efficacy and network stability, although direct TI-specific evidence remains limited [132,133,134,135].

Building on these mechanisms, it is also possible that activity-dependent changes in brain activity resulting from stimulation may influence adult neurogenesis, particularly in the hippocampus, although direct evidence for TI-induced neurogenesis remains limited [136]. This brain region is critical for memory and emotional regulation, and newly generated neurons here contribute to cognitive flexibility. Although direct experimental evidence for TI-induced neurogenesis remains limited, these cellular processes represent promising avenues for future mechanistic research. Determining how stimulation affects neurogenesis could lead to targeted interventions to enhance cognitive function or support rehabilitation [137,138]. These mechanisms should currently be regarded as plausible hypotheses or extrapolations from related neuromodulation research, rather than effects that have been conclusively demonstrated for TI stimulation itself.

Although these findings suggest potential biological mechanisms underlying TI-induced neuromodulation, further research is still needed to define the optimal stimulation parameters, including waveform, intensity, and frequency [20,24]. TI also offers a non-invasive approach for steerable modulation of deep brain electric fields, with the potential to improve targeting specificity and to support safer application through biophysical and dosimetric optimization [18,100].

### 4.5. Potential Gene Expression and Epigenetic Regulatory Basis of Long-Term Therapeutic Effects

TI stimulation may produce effects that persist beyond the stimulation period [97,139]. Such effects may not be restricted to immediate electrophysiological changes, but could also involve longer-term plasticity-related molecular and cellular processes [118]. Evidence from other neuromodulation studies and from research on activity-dependent neural plasticity further suggests that transcriptional regulation and epigenetic mechanisms may participate in long-term neural reorganization, providing a possible basis for understanding the sustained therapeutic effects of TI [140,141].

In addiction research, ΔFosB and cAMP response element-binding protein (CREB) are widely regarded as key transcriptional regulators, whereas BDNF is considered an important neurotrophic regulator involved in behavior-related neural plasticity and pathological reward memory [141,142]. Given emerging evidence that TI can modulate craving-related neural processes, it has been proposed that TI may indirectly influence addiction-related transcriptional and neurotrophic pathways by altering the functional states of reward-related circuits, although this possibility remains hypothetical and has not yet been directly demonstrated [139]. Together, these considerations suggest that TI may affect not only immediate behavioral responses, but may also hold more sustained therapeutic potential for addiction through transcription-dependent plasticity mechanisms [140,141].

Possible links between temporal interference (TI) stimulation and epigenetic regulation have begun to attract increasing attention; however, this relationship remains largely hypothetical and has not yet been directly demonstrated in TI-specific studies [143]. Evidence from activity-dependent neural plasticity and other neuromodulation approaches suggests that sustained changes in neuronal activity can induce epigenetic modifications. Previous studies have shown that alterations in histone deacetylase (HDAC) activity and remodeling of DNA methylation patterns can regulate chromatin accessibility, thereby influencing the transcription of genes related to emotion and motivation [144]. Given that TI stimulation may induce sustained modulation of neural activity and synaptic plasticity, it is plausible that similar activity-dependent epigenetic mechanisms may be engaged, thereby contributing to long-term neural network remodeling. This hypothesis is particularly relevant in the context of mood disorders, such as de-pression, where inhibition of HDAC activity has been shown to promote chromatin relaxation and produce antidepressant-like effects, as well as enhance stress-related behavioral adaptation [144,145]. Nevertheless, it should be emphasized that direct experimental evidence linking TI stimulation to epigenetic modifications is currently lacking, and further studies are required to determine whether such mechanisms indeed underlie its long-term neuro-modulatory effects.

In studies of neurodegenerative diseases, the long-term effects of TI stimulation may similarly involve the remodeling of gene-expression profiles. Evidence from the broader neurodegeneration literature suggests that inflammatory processes, including pro-inflammatory mediators such as interleukin-1β (IL-1β) and tumor necrosis factor-α (TNF-α), are closely involved in disease progression [146,147]. In parallel, BDNF-related signaling is strongly associated with neuroprotection, synaptic homeostasis, and the maintenance of neural network stability [147,148]. Against this background, TI stimulation may theoretically influence these pathways through mechanisms analogous to those reported in other neuromodulation paradigms. However, direct TI-specific evidence demonstrating reduced inflammatory mediators or enhanced BDNF-related signaling in neurodegenerative disease models remains limited. Taken together, these considerations suggest a plausible—though still unconfirmed—molecular basis for the potential long-term effects of TI on cognitive improvement and the slowing of disease progression [120].

Although the gene expression and epigenetic regulatory mechanisms potentially associated with TI stimulation are still being explored, current evidence supports the possibility that such molecular changes could contribute to the sustained therapeutic effects of TI [97,118,139]. By analogy with other activity-dependent and non-invasive stimulation paradigms, TI may allow neuromodulatory interventions to extend from transient electrophysiological regulation toward more persistent molecular and structural remodeling of the nervous system [140].

Multilevel regulation from neural oscillations to molecular plasticity may constitute a plausible mechanistic framework underlying the potential long-term therapeutic effects of TI stimulation [118]. Although TI-induced changes in gene expression and their epigenetic regulation are still under investigation, current studies indicate that such molecular-level alterations may provide a biological basis for the sustained functional improvements observed after TI stimulation [97,118]. By influencing neural activity over behaviorally relevant timescales, TI may engage downstream plasticity mechanisms that extend beyond immediate electrophysiological modulation, thereby supporting its potential long-term therapeutic value across chronic brain disorders [140].

### 4.6. Safety Profile of Temporal Interference Electrical Stimulation

Current evidence indicates that TI stimulation demonstrates a favorable safety profile in both experimental and clinical contexts [39]. By enabling precise modulation of deep brain regions through low-frequency envelope electric fields, TI largely avoids unintended stimulation of superficial cortical areas and is generally well tolerated. Using c-fos immunostaining as an indicator of neuronal activation, experimental studies have shown that TI selectively activates deep neural structures, including the hippocampus, while leaving superficial cortical regions largely unaffected [18]. Moreover, available preclinical evidence has not identified gross histopathological abnormalities under the tested TI conditions, including no obvious neuronal loss or marked glial reactivity, although more systematic evaluation of apoptosis-, DNA damage-, and neuroinflammation-related markers remains warranted [149].

In addition, TI stimulation does not appear to produce substantial thermal effects under the tested experimental conditions, as prior work reported only minimal increases in brain temperature during stimulation. Taken together, these findings provide preliminary support for the short-term safety of TI as a noninvasive neuromodulation approach under the tested experimental conditions [18].

TI-related stimulation paradigms can also be implemented using repurposed clinically approved hardware, and flexible conformable on-skin multielectrode arrays may facilitate spatial alignment and targeting, at least in peripheral nerve applications [150]. In peripheral nerve models, temporally interfering stimulation achieved effective actuation at lower current amplitudes than standard transcutaneous electrical stimulation, suggesting potential advantages in depth and efficiency over conventional transcutaneous approaches [150].

However, current studies indicate that the mechanism of TI stimulation cannot be explained solely by low-pass filtering at the neuronal membrane, but instead involves nonlinear rectification mediated by ion channels. While this rectification allows neuronal responses to low-frequency envelope modulation, it may also induce high-frequency conduction block in non-target regions, which has implications for clinical application. Accordingly, although TI shows promise for treating disorders involving deep brain structures, stimulation design should consider potential conduction block effects and be supported by appropriate experimental validation [36].

## 5. Comparison of Temporal Interference Electrical Stimulation with Other Brain Stimulation Techniques

Brain stimulation techniques currently used in the modulation of neuropsychiatric disorders include transcranial magnetic stimulation (TMS), transcranial electrical stimulation (tES), and invasive DBS, each with distinct advantages and limitations in stimulation principles, depth of action, and clinical accessibility. As research increasingly emphasizes precise intervention at the level of deep neural circuits and distributed networks, the need for approaches that combine non-invasive delivery with effective deep brain targeting has become more evident. TI stimulation, which employs envelope modulation generated by the vector superposition of electric fields, provides a potential technological pathway linking conventional non-invasive stimulation methods with invasive deep brain stimulation [18,19].

### 5.1. Comparison with TMS

TMS modulates neuronal excitability by inducing electric currents in cortical tissue through rapidly changing magnetic fields and is one of the most established non-invasive neuromodulation techniques currently in use [151]. TMS has been widely applied in the study and treatment of neuropsychiatric disorders, with reported effects in depression, addiction, obsessive–compulsive disorder, and certain cognitive impairments [152]. The left dorsolateral prefrontal cortex (DLPFC) is a commonly targeted region, where stimulation is thought to improve clinical symptoms by enhancing cognitive control, regulating emotional processing, and suppressing maladaptive motivational drive [152,153].

However, the physical properties of TMS inherently limit its effects to relatively superficial cortical regions. The effective stimulation depth of conventional coils is generally restricted to the cortex, typically within 2–3 cm, making direct modulation of deep brain structures such as the nucleus accumbens, thalamus, basal ganglia, and limbic system difficult, despite their important roles in many neuropsychiatric disorders [154]. In addition, higher stimulation intensities may induce headaches, facial muscle twitching, or discomfort, and the spatial precision of TMS is constrained by coil geometry and placement, limiting its capacity for accurate deep modulation at the level of complex brain networks [155].

In contrast, TI stimulation employs high-frequency carrier signals to generate low-frequency difference-envelope fields within the brain, allowing relatively focused stimulation of deep brain regions while remaining non-invasive [18]. This feature enables modulation of thalamocortical, basal ganglia-cortical, and limbic system-related circuits, with comparatively limited effects on non-target cortical areas and good overall tolerability. Rather than replacing TMS, TI complements conventional cortex-focused neuromodulation strategies by extending non-invasive intervention to deep neural networks, thereby showing potential value for precise modulation in a range of neuropsychiatric disorders.

### 5.2. Comparison with tES

Transcranial electrical stimulation (tES), including transcranial direct current stimulation (tDCS) and transcranial alternating current stimulation (tACS), applies weak electrical currents to the scalp to modulate cortical neuronal excitability or synchronize neural oscillations within specific frequency bands [156]. In recent years, tES has been widely used as a non-invasive neuromodulation approach in cognitive neuroscience and neuropsychiatric research. Previous studies suggest that tES can influence neuronal membrane polarization and synaptic plasticity, and meta-analytic evidence indicates that its effects may be particularly evident when stimulation is combined with learning or behavioral training, especially in language- and numerical-learning contexts [157].

In neuropsychiatric disorders, tES has been explored as an intervention in conditions including depression, addiction, schizophrenia, and mild cognitive impairment [158]. By modulating excitability in the prefrontal cortex and associated networks, tES has shown potential for improving emotional regulation, reducing maladaptive craving, and enhancing cognitive control. However, substantial heterogeneity in stimulation parameters, target selection, and efficacy evaluation, together with pronounced inter-individual variability, remains a major limitation for its clinical translation and standardized application [159].

From a technical perspective, the electric fields generated by conventional tES are relatively diffuse and are strongly shaped by anatomical factors such as skull and tissue properties as well as by electrode montage, which limits spatial selectivity and makes modulation of deeper structures less direct and less controllable than cortical modulation [160,161]. In contrast, TI stimulation employs low-frequency difference-envelope fields to generate more focused electric fields within the brain, enabling relatively precise modulation of deep regions while remaining non-invasive and minimizing effects on non-target cortical areas. As such, TI may partially address the limitations of conventional tES related to stimulation depth and spatial precision, and shows promise for deep circuit modulation and individualized intervention [17].

### 5.3. Comparison with DBS

DBS is currently the most established invasive neuromodulation technique for the modulation of deep brain regions and is widely used in clinical practice for movement disorders such as Parkinson’s disease, essential tremor, and dystonia; it has also received regulatory approval or humanitarian authorization in specific contexts for severe obsessive–compulsive disorder and drug-resistant epilepsy, whereas its application in major depressive disorder remains investigational [162,163]. Depending on the target selected—such as the subthalamic nucleus (STN), the globus pallidus internus (GPi), the thalamus, or the nucleus accumbens (NAc)—DBS can modulate basal ganglia–thalamo–cortical and limbic circuits, thereby improving motor symptoms and, in selected neuropsychiatric indications, compulsive or affective symptoms [163,164].

Although DBS demonstrates substantial therapeutic efficacy, it does so at the cost of high invasiveness. The technique requires electrode implantation via a craniotomy and long-term reliance on an implanted pulse generator, with associated risks including infection, hemorrhage, electrode displacement, and hardware-related complications [165,166]. In addition, the need for repeated postoperative programming, long-term follow-up, and the associated logistical and financial burden further limits its application in broader patient populations [167,168]. As a result, reducing invasiveness while preserving the advantages of deep brain modulation has become an important direction in the development of neuromodulation technologies.

In this context, TI stimulation has been proposed as a novel non-invasive deep-targeting neuromodulation strategy. By applying multiple high-frequency carrier currents, TI generates a low-frequency difference-envelope field within the brain, offering the possibility of relatively selective modulation of deep structures without surgical implantation [19,32]. Rather than being considered a replacement for DBS at present, TI is more appropriately viewed as a promising bridge between conventional non-invasive stimulation and invasive deep brain stimulation. With continued advances in electric-field modeling, stimulation-parameter optimization, and individualized targeting, TI may expand the range of non-invasive interventions for deep neural circuits, although its efficacy and clinical utility still require further validation [17,100].

Overall, TI stimulation has been proposed to offer several potential advantages as a non-invasive deep-targeting neuromodulation approach. Evidence from computational modeling and an early human hippocampal study supports the feasibility of relatively selective deep targeting while reducing stimulation of overlying cortex [32]. Preliminary human data also suggest that TI can be tolerated under tested conditions [169]. However, systematic safety characterization and application standards are still evolving [100].

### 5.4. Comparison with tFUS

In addition to commonly used electrical and magnetic stimulation techniques, emerging non-invasive approaches, such as transcranial focused ultrasound stimulation (tFUS), have been explored for deep brain modulation. tFUS has attracted increasing attention for its potential to modulate deep brain structures [170]. Compared with conventional transcranial stimulation approaches, tFUS provides relatively high spatial precision, with reported millimeter-scale resolution (approximately 1–5 mm), and strong penetration capability for subcortical targets. These characteristics make tFUS an important reference technique for evaluating the spatial precision and depth penetration of Temporal Interference (TI) Electrical Stimulation, especially when considering their relative advantages for non-invasive deep brain modulation [171]. In addition, tFUS has been investigated across a range of neurological and psychiatric conditions, and its neuromodulatory effects have been associated with multiscale mechanisms involving ion channels, synaptic transmission, neural oscillations, and large-scale brain networks [172].

In contrast to tFUS, which serves as a benchmark for spatial precision and depth, TI relies on the interference of high-frequency electric fields and may present a different approach, offering flexibility and potential accessibility for non-invasive deep neuromodulation [37]. In particular, TI enables adjustable depth selectivity through electrode configuration, current balancing, and frequency tuning. It can also be readily integrated with existing electrical stimulation platforms, individualized electric-field modeling, and multimodal monitoring approaches [32]. However, direct experimental evidence comparing the spatial focality of TI with tFUS is currently limited. Transcranial focused ultrasound (tFUS) has been shown to achieve millimeter-scale spatial resolution and precise depth targeting by focusing acoustic energy deep into the brain, surpassing the spatial specificity of many conventional non-invasive stimulation methods, such as TMS and electrical stimulation techniques. While TI uses high-frequency electric field interference to modulate deep regions, its achievable spatial focality may be inherently constrained by factors such as skull-related current shunting and tissue conductivity. These theoretical and computational considerations suggest that TI’s spatial precision could be lower than tFUS under some conditions, but direct comparative experimental data remain sparse. Therefore, further comparative studies are needed to directly assess the spatial focality and depth selectivity of TI relative to tFUS [173].

At the same time, tFUS faces several significant technical and translational challenges. Its neuromodulatory effects are influenced by multiple acoustic parameters, such as fundamental frequency, acoustic pressure, pulse duration, pulse repetition frequency, duty cycle, and pulse train duration [170]. Moreover, the human skull significantly attenuates and distorts ultrasound transmission, often reducing acoustic intensity by 65–90% at typical neuromodulation frequencies. Although low-intensity tFUS is designed to avoid significant thermal injury, questions regarding long-term safety, optimal parameter selection, auditory side effects, and protocol standardization remain unresolved [171].

Taken together, TI and tFUS may be considered complementary rather than competing approaches [18]. tFUS offers advantages in ultrafocal targeting of deep brain structures. In contrast, TI provides a non-invasive electrical approach that enables adjustable deep neuromodulation and facilitates integration with existing stimulation and modeling frameworks [20]. Given these advantages, both techniques are promising. However, further optimization, safety validation, and disorder-specific clinical studies are essential for broader translational application [174].

### 5.5. Clinical Accessibility and Cost-Effectiveness

**Figure 4:**
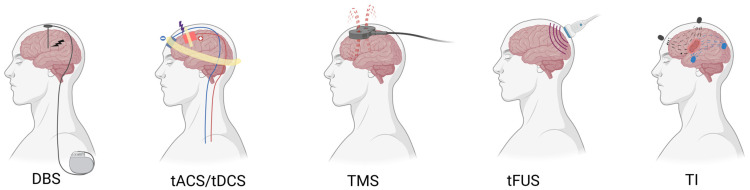
Representative schematics of major brain stimulation modalities.

Clinical accessibility ultimately influences the adoption of neuromodulation technologies. Although TI currently requires advanced hardware and individualized modeling, it may offer future advantages in accessibility and long-term cost structure compared with invasive neurosurgical approaches, particularly if hardware, targeting workflows, and stimulation protocols become standardized [175]. Unlike TMS, which often entails repeated clinic-based sessions, TI may eventually provide a scalable option for deep brain modulation as stimulation hardware and implementation pathways continue to evolve [30,176]. Representative schematics of major brain stimulation modalities are shown in Figure 4, and a comparative summary of their key characteristics is provided in Table 1.

## 6. Research Prospects of Temporal Interference Electrical Stimulation in Human Neuromodulation

### 6.1. Limitations and Technical Challenges of Temporal Interference Electrical Stimulation

Despite its considerable promise, temporal interference electrical stimulation (TI) faces several significant limitations and technical challenges. A key challenge is the complexity of achieving precise deep-target engagement [24]. Although TI is designed to generate constructive interference in deep brain regions while minimizing stimulation of the overlying cortex, reliable targeting in practice remains highly dependent on precise electrode placement, current balancing, and individual anatomical characteristics [177]. Differences in skull thickness, tissue conductivity, head geometry, and electrode positioning can significantly affect the spatial distribution of the electric field, leading to considerable inter-individual variability in stimulation outcomes [177]. Consequently, ensuring selective stimulation of deep brain structures, such as the amygdala, while minimizing effects on surrounding regions remains an important challenge [178].

Another challenge involves balancing efficacy, safety, and tolerability. TI generally operates at low intensities, thereby avoiding substantial tissue heating [20]. However, higher stimulation intensities may increase the strength of the generated electric field in deep targets while simultaneously increasing cutaneous (skin) discomfort, creating a practical trade-off between effective neuromodulation and tolerability [179]. Additionally, factors like electric-field asymmetry, amplitude steering, and current imbalance can shift the stimulation focus, making parameter optimization more challenging and raising the risk of off-target effects [120].

Methodological challenges also arise in monitoring TI-induced neural activity. High-frequency stimulation currents may introduce artifacts (unintended signals) in electrophysiological recordings or neuroimaging signals, potentially interfering with EEG (electroencephalography) or MRI (magnetic resonance imaging) measurements. Because TI relies on the generation of a low-frequency envelope (a slowly varying signal created by the interaction of high-frequency currents), careful experimental design and signal-processing strategies are required to separate stimulation artifacts from genuine neural signals. Recent neuroimaging studies have suggested that TI can selectively modulate task-relevant neural circuits without inducing large-scale alterations in the global brain network topology (the overall organization of brain connections). For example, theta-band (4–8 Hz) TI stimulation targeting the hippocampus has been shown to selectively enhance hippocampus-related functional connectivity (coordinated activity between brain regions) following memory tasks, while leaving whole-brain network organization largely unchanged. These findings suggest that TI may modulate specific neural circuits while producing relatively limited interference with neuroimaging measurements [180].

In addition to these technical complications, another significant limitation is the variability in stimulation parameters. Currently, optimal protocols for frequency, amplitude, and duration remain unstandardized. Experimental studies have used current intensities ranging from approximately 0.25 mA to 2 mA and carrier frequencies from 100 Hz to 2 kHz [149]. Differences in these parameters may substantially influence stimulation depth, electric-field distribution, and neuromodulatory outcomes, which may partly explain the variability observed across studies [181].

To address these challenges, future research should prioritize individualized optimization strategies. Personalized electric-field modeling, tailored to individual anatomical features, may improve targeting precision and minimize off-target stimulation. Additionally, optimizing electrode configurations and current ratios (e.g., 1:4 to 4:1) could further refine the electric field and enhance selective modulation of deep brain structures. Developing standardized stimulation protocols, improving artifact-management techniques, and implementing multimodal validation approaches will be essential for advancing the clinical translation of TI stimulation [34].

### 6.2. Safety and Potential Side Effects of Temporal Interference Electrical Stimulation

Safety is a key aspect of TI stimulation. Experimental evidence shows that TI uses low-intensity electrical currents that remain well below thresholds for tissue damage or thermal effects [169]. Computational models and animal studies show that TI-generated fields do not heat tissue or damage neuronal membranes at standard stimulation settings [97].

Despite these encouraging findings, important uncertainties remain. If stimulation parameters are not precisely controlled, TI could theoretically alter synaptic plasticity or promote abnormal neuronal synchronization. However, no consistent evidence has demonstrated TI-induced epileptiform activity in the available experimental literature. Nevertheless, systematic safety assessments remain limited [20,182]. In addition, preliminary human work has shown no significant differences in EEG measures, neuropsychological performance, or overt adverse effects between active TI-tACS and sham conditions at typical intensities (≈2 mA, 20/70 Hz, 30 min). Taken together, these findings support short-term tolerability and the absence of acute abnormal EEG activity under these conditions, although larger and more detailed electrophysiological studies are still needed [169].

Importantly, most existing studies have been conducted in small cohorts or within short-term experimental settings. As a result, the long-term safety, cumulative effects of stimulation, and potential interactions with pre-existing neurological conditions still require thorough evaluation. Future research should include larger clinical samples, extended follow-up periods, and comprehensive neurophysiological monitoring to better establish the safety profile of TI stimulation [57].

### 6.3. Future Directions and Translational Prospects

Although research on TI stimulation in humans remains at an early exploratory stage, existing experimental studies suggest its potential value in the deep modulation of emotional regulation, reward processing, and cognitive control [114,183]. By enabling targeted modulation of deep brain regions while maintaining a non-invasive approach, TI provides a new technical pathway for individualized deep-brain neuromodulation and clinical neurotherapeutics [20].

Mechanistic investigations of TI stimulation have increasingly incorporated BOLD-fMRI together with individualized structural imaging and three-dimensional electric field simulations, allowing dynamic assessment of stimulation-related changes in brain activity and optimization of electrode configurations for deep-brain targeting [32,183,184]. These approaches provide objective measures of target engagement and functional modulation in deep neural circuits, while MRI-compatible TI systems have also shown favorable safety and feasibility profiles, supporting future translational studies that combine TI with multimodal monitoring strategies [32,183].

In summary, although existing neuromodulation approaches have demonstrated therapeutic benefits across a range of brain disorders, they remain limited by insufficient stimulation depth, restricted targeting precision, or the risks associated with invasive procedures. As a novel non-invasive deep-brain modulation technique, TI stimulation enables relatively precise regulation of deep brain structures and their associated functional networks while maintaining a favorable safety profile [20,32]. These characteristics support its potential applicability in movement disorders, cognitive impairment, addiction, and other brain disorders [20,81]. Nevertheless, critical issues-including stimulation parameter optimization across disease states, inter-individual variability, and the long-term neurobiological consequences of TI-have yet to be systematically investigated. Addressing these challenges will be essential for advancing mechanistic understanding and facilitating the clinical translation of TI-based neuromodulation.

## 7. Conclusions

TI stimulation is an emerging non-invasive deep neuromodulation technique that shows potential for relatively targeted regulation of deep brain structures and their associated functional networks. In recent years, it has gradually attracted attention as a potentially important methodological direction in neuropsychiatric research and intervention. Existing studies suggest that the modulatory effects of TI may involve multiple levels of neural regulation, including the modulation of pathological neural oscillations, changes in deep-cortical functional connectivity, adjustment of excitatory-inhibitory balance, and plasticity-related neural reorganization. These neural changes may further be accompanied by downstream molecular responses, thereby forming a potential modulatory framework extending from electrophysiological regulation to network- and molecular-level remodeling. On this basis, TI may hold promise for application across a range of neuropsychiatric disorders, including mood disorders, cognitive impairment, movement disorders, epilepsy, and addiction, and may provide a useful complement to existing therapeutic strategies.

Nevertheless, the clinical translation of TI stimulation in neuropsychiatric disorders remains at an early stage. Several fundamental issues, including the optimization of stimulation parameters, the influence of individual anatomical and functional variability on electric field distribution and treatment response, and the long-term safety and durability of therapeutic effects, still require further investigation. Future studies will need to integrate high-resolution computational modeling with multimodal neuroimaging, as well as closed-loop or adaptive stimulation approaches, in order to support more precise and individualized intervention strategies. Overall, the continued development of TI may contribute to more refined and network-oriented deep brain intervention within the field of non-invasive neuromodulation, and may offer new perspectives for both mechanistic research and clinical therapy in neuropsychiatric disorders.

## Figures and Tables

**Table 1:** Comparison of Brain Stimulation Modalities.

Modality	DBS	tES (tDCS/tAC)	TMS	tFUS	TI
Invasiveness	Invasive	Non-Invasive	Non-Invasive	Non-Invasive	Non-Invasive
Stimulation depth	Deep	Superficial	Superficial	Deep	Deep
Spatial focality	High	Low	Moderate	High	Moderate
Advantages	Precise modulation	Simple, portable, low cost	Clinically effective	High precision	Non-invasive deep
Limitations	Surgery risks	Diffuse fields, variability	Limited penetration	Safety	Safety, complexity
Clinical Applications	Pain, Parkinson’s, Tremor, Movement disorders	Pain, Depression, Cognitive enhancement, Mood disorders	Depression, Pain, Anxiety, OCD, Stroke rehabilitation	Pain, Neurological conditions, Brain mapping	Depression, Cognitive disorders, Memory enhancement
Effectiveness	Long-term, continuous stimulation	Short sessions (20–30 min), daily or few times per week	20–40 min per session, few times per week	Short sessions (5–30 min), frequency depends on condition	Short, intermittent sessions (10–20 min), needs further validation
Side Effects	Infection, headache, bleeding, hardware malfunction	Skin irritation, headache, dizziness, fatigue	Scalp discomfort, headache, transient hearing effects	Skin irritation, discomfort at stimulation site	Mild headache, fatigue, dizziness

Abbreviations: DBS = deep brain stimulation; tES = transcranial electrical stimulation (including tDCS and tACS); tDCS = transcranial direct current stimulation; tACS = transcranial alternating current stimulation; TMS = transcranial magnetic stimulation; tFUS = transcranial focused ultrasound stimulation; TI = temporal interference **electrical** stimulation.

## Data Availability

No new data were created or analyzed in this study. Data sharing is not applicable to this article.

## Abbreviations

The following abbreviations are used in this manuscript:

TI	Temporal Interference
FEM	Finite element model
NAc	Nucleus accumbens
VTA	Ventral tegmental area
MDD	Major depressive disorder
PTSD	Post-traumatic stress disorder
mPFC	medial PFC
DBS	Deep-brain stimulation
PFC	Prefrontal cortex
PD	Parkinson’s disease
STN	Subthalamic nucleus
GPi	Globus pallidus
DLS	Dorsolateral striatum
CPP	Conditioned place preference
DA	Dopaminergic
E/I	Excitatory/inhibitory
AD	Alzheimer’s disease
MCI	Mild cognitive impairment
DMN	Default mode network
DRE	Drug-resistant epilepsy
BDNF	Brain-derived neurotrophic factor
tACS	Transcranial alternating current stimulation
tDCS	Transcranial direct current stimulation
GABA	γ-aminobutyric acid
CREB	cAMP response element-binding protein
HDAC	Histone deacetylase
TENS	Transcutaneous electrical nerve stimulation
TMS	Transcranial magnetic stimulation
TES	Transcranial electrical stimulation
DLPFC	the left dorsolateral prefrontal cortex
